# Closing in on the constitution of consciousness

**DOI:** 10.3389/fpsyg.2014.01293

**Published:** 2014-11-17

**Authors:** Steven M. Miller

**Affiliations:** ^1^Monash Alfred Psychiatry Research Centre, Monash University Central Clinical School and The Alfred Hospital, Melbourne, VIC, Australia; ^2^School of Psychological Sciences, Monash University, Melbourne, VIC, Australia

**Keywords:** consciousness, neural correlates, constitution, binocular rivalry, philosophy of mind, mechanistic explanation, foundations, philosophy of science

## Abstract

The science of consciousness is a nascent and thriving field of research that is founded on identifying the minimally sufficient neural correlates of consciousness. However, I have argued that it is the neural constitution of consciousness that science seeks to understand and that there are no evident strategies for distinguishing the correlates and constitution of (phenomenal) consciousness. Here I review this correlation/constitution distinction problem and challenge the existing foundations of consciousness science. I present the main analyses from a longer paper in press on this issue, focusing on recording, inhibition, stimulation, and combined inhibition/stimulation strategies, including proposal of the Jenga analogy to illustrate why identifying the minimally sufficient neural correlates of consciousness should not be considered the ultimate target of consciousness science. Thereafter I suggest that while combined inhibition and stimulation strategies might identify some constitutive neural activities—indeed minimally sufficient constitutive neural activities—such strategies fail to identify the whole neural constitution of consciousness and thus the correlation/constitution distinction problem is not fully solved. Various clarifications, potential objections and related scientific and philosophical issues are also discussed and I conclude by proposing new foundational claims for consciousness science.

## HISTORICAL CONTEXT OF THE PROBLEM

The science of consciousness is founded on searching for the neural correlates of consciousness (Crick and Koch, 1990, 1998; Crick, 1994; usually referred to as “NCC” but here as “NCrC,” unless quoting directly). Specifically, consciousness science seeks to identify the *minimally sufficient* NCrC. Chalmers (2000) provided a foundational work concerning this notion, in a paper entitled, “What is a neural correlate of consciousness?” His paper is widely cited, as is his definition of the NCrC (p. 31):

An NCC is a minimal neural system N such that there is a mapping from states of N to states of consciousness, where a given state of N is sufficient, under conditions C, for the corresponding state of consciousness.

Also widely cited is the shorter definition by Koch (2004, p. 16) in which the NCrC is considered to be the “minimal set of neuronal events and mechanisms jointly sufficient for a specific conscious percept.” Although there has been some discussion of Chalmers’ definitions and claims, and the methodological assumptions on which they are based (e.g., Noë and Thompson, 2004; Bayne, 2007; Hohwy, 2007, 2009; Neisser, 2012; Bayne and Hohwy, 2013), there has been less scrutiny than might be expected for a foundational work in a nascent scientific discipline.

A few years ago, two groups of scientists (Aru et al., 2012; de Graaf et al., 2012) independently parsed NCrCs into “NCrC substrate” or “NCrC proper” on the one hand (the construct of interest for consciousness science) and “NCrC precursors/prerequisites” and “NCrC consequences” on the other (constructs of lesser interest). Their partition was aimed at developing strategies to distinguish these various NCrCs and this is of course, the topic of this special issue. In proposing this terminology, however, these authors were unaware that the problem of identifying which, from among many, NCrCs^1^ are most directly relevant to consciousness had been appreciated, named and analyzed a decade prior.

Both Revonsuo (2000, 2001, 2006, in press) and myself (Miller, 2001, 2007, 2013a, in press-a,b) have been explicitly concerned with just how science will distinguish the neural *correlates* and the neural *constitution* of consciousness (NCnC). This is particularly problematic for *phenomenal* consciousness—the subjective or qualitative nature of our conscious states (Nagel, 1974). Revonsuo’s (2000, 2001) concern was that current scientific methodologies—brain recording techniques in particular—are not capable of targeting the right level of organization in the brain (which he termed, “the phenomenal level”). Revonsuo (2006, in press) also developed a highly detailed level-based biological framework for a consciousness research program that seeks to discover the *constitutive mechanisms* of consciousness.

Although I have a great deal of affinity with Revonsuo’s concerns and his framework, my development of the issue has been different. I noted (Miller, 2001) that although the problem of identifying the constitution of consciousness had been *alluded to* by investigators in the science of consciousness, such as Francis Crick, Christof Koch, and Nikos Logothetis (Crick, 1994; Crick and Koch, 1998; Logothetis, 1998)^2^, it required much more than such passing reference. I therefore sought to name and examine the *correlation/constitution distinction problem* (herewith, Cr/Cn distinction problem) so that it might be addressed *explicitly*, with a view to its solution or dissolution, or indeed to its acceptance as an ultimate epistemic limit.

I explicated the Cr/Cn distinction problem utilizing the phenomenon of binocular rivalry (Miller, 2001). Unlike Revonsuo, my initial analysis led me to be concerned that perhaps even future scientific methodologies, including those targeting the right level of organization in the brain, might fail to solve the problem. I suggested there were no obvious corollaries regarding the NCnC from studies of the NCrC and yet it was the NCnC that a science of consciousness should ultimately wish to identify. I therefore proposed that consciousness science might require entirely new scientific strategies if it is to move from mere correlation to actual constitution. My initial concerns were re-stated and developed in a subsequent paper (Miller, 2007), in which I noted (p. 161, italics in original):

If we imagine that through the employment of all current and future neuroscientific methods (in all contexts, under all conditions and with all methodological constraints overcome), we were able to obtain a complete, real-time and multimodal description of all the NCC and all observable properties of such, would we be satisfied that we had obtained a comprehensive understanding of the neuroscience of consciousness? I assert not, because *not every neural correlate of a conscious state is necessarily constitutive of that state.*

In both my 2001 and 2007 papers, I discussed how NCrC recording, inhibition (disablement), and stimulation techniques—either alone or in combination—failed to distinguish correlated but non-constitutive neural activities that are *upstream* or *downstream* from correlated constitutive neural activities (Miller, 2001, 2007). (This upstream/downstream terminology is equivalent to the precursors/consequences terminology and is also used by others; e.g., Chalmers, 2000; Hohwy and Bayne, in press.)^3^ I considered therefore, that these empirical approaches failed to yield conclusions regarding the NCnC, that entirely new approaches might be needed and that the Cr/Cn distinction problem might need to join other well-known hard problems of consciousness. I also made it clear, however, that there were no grounds, as yet, to proclaim the problem intractable (Miller, 2007).

## CURRENT STATUS OF THE PROBLEM

Discussion of the Cr/Cn distinction problem has since gained momentum, evidenced by this special issue and by a two-volume project in which scientists and philosophers discuss the problem and the conceptual, empirical, and philosophical territory within which it is situated. In the first volume (Miller, 2013a), scientific groundwork was laid with papers on the brain and visual system’s constituents, organization and processes, on the current status of binocular rivalry research from multiple empirical perspectives and on current neuroscientific investigative techniques (including various invasive and non-invasive recording, inhibition, and stimulation techniques).

The second volume (Miller, in press-a) addresses scientific and philosophic perspectives on consciousness science and its methods and foundational constructs, the Cr/Cn distinction problem, the philosophical territory of phenomenal consciousness, hard problems of consciousness, the notion of explanation in consciousness science, the relation between brain and mind, and in particular, notions of correlation, constitution, identity, causation, supervenience, emergence, and realization. My paper in that second volume (Miller, in press-b) closely examines Chalmers’ (2000) foundational notion of the minimally sufficient NCrC and through that analysis suggests new foundational claims for consciousness science.

In the present paper—given the topic of this special issue—I outline the specifically *methodological* aspects from Miller (in press-b). I start by providing brief reference to foundational issues in consciousness science and then discuss neural inhibition approaches that challenge those issues. Thereafter I discuss neural stimulation and the combined inhibition/stimulation approach, suggesting that although the latter may provide the best evidence for identifying at least some constitutive neural activities, it fails to fully solve the Cr/Cn distinction problem. Various points of clarification are then discussed and six objections to the presented arguments are listed, with three discussed in detail. A brief presentation of related scientific and philosophic issues is then provided and I conclude with the proposed new foundational claims for consciousness science.

## A BRIEF SCAN OF THE FOUNDATIONS

The Cr/Cn distinction problem is neatly exposed when considering NCrCs during binocular rivalry. This visual phenomenon—in which *dynamic* perceptual alternations are induced by *static* presentation of a different image to each eye—provides several advantages for the scientific study of consciousness (reviewed in Miller, 2013b). In particular, binocular rivalry allows for dissociation between neural activity correlated with a subject’s perceptual alternations and neural activity correlated with image presentation. Thus, *perception-dependent* neural activity is rightly considered a neural correlate of visual consciousness during rivalry. However, the perception-dependent data for rivalry from electrophysiological and brain-imaging *recording* studies (reviewed in many chapters in Miller, 2013a) yield a wide array of such NCrCs. This *makes evident* the Cr/Cn distinction problem. That is, because not every NCrC is necessarily constitutive of that conscious state, we can ask *which* NCrCs from this array are actually constitutive. Moreover, we can ask what methodologies science might employ to experimentally examine various hypotheses in this regard.

In Chalmers’ (2000) examination of the NCrC notion, he discusses the range of cases over which a correlation should be expected to hold. Those discussed include the normal brain, unusual input (such as binocular rivalry), lesion studies, and stimulation studies. Chalmers’ analysis is predominantly conceptual rather than a detailed methodological approach to identifying the NCrC, but he does discuss the lesion case in some depth. He notes that such studies should be regarded very cautiously and perhaps abandoned altogether in searching for the NCrC, due to the altered brain architecture they induce (thus suggesting that an NCrC should be architecture-dependent not architecture-independent)^4^. Chalmers discusses in far less detail, the methodological NCrC approach to the normal brain, unusual input, and brain stimulation. He notes nonetheless that there are interpretive complexities with unusual input and brain stimulation too, but he considers these cases and the normal brain case to be those over which an NCrC should be required to hold (with perhaps some “good” lesion approaches also being admitted). The present analyses aim to address in detail, inhibition and stimulation empirical strategies—and their interpretive complexities—that surround the notion of the minimally sufficient NCrC.

There are several other aspects to Chalmers’ (2000) formulation of the NCrC construct that are worth mentioning. First, his definition is intended to constrain the discussion to “correlation” terminology, as he considers this to be theoretically neutral rather than theoretically loaded. Second, he notes his definition is constructed in a way that provides a tractable methodology for NCrC identification, and thus a way forward for consciousness science. Third, he accepts that even if the thus-defined NCrC was identified, this would not necessarily *explain* consciousness and may not even be the key to understanding processes underlying consciousness. However, he does not *explicitly* consider the potential for a Cr/Cn distinction problem, perhaps because of his reluctance to shift the discussion beyond notions of correlation. Moreover, although his formulation of the minimally sufficient NCrC construct acknowledges the empirical potential it provides, it does not adequately examine the empirical entailment of the “minimally sufficient” qualifier or the methodological details of such entailment.

As outlined in greater detail in Miller (in press-b), despite the valuable contribution provided by Chalmers’ conceptual proposals, there are several problems with accepting his formulation as unchallenged foundations for consciousness science. Indeed, Chalmers himself considered his work to be “conceptual spadework” that would require refinement. If my contention above is accurate—that consciousness science wishes to ultimately identify the NCnC—then it is noteworthy that this construct is nowhere to be seen in Chalmers’ analysis. It is a construct that can be conveyed using a wide range of terms such as the neural “basis,” “mechanism,” or “substrate” of consciousness (see Miller, 2007 for more terms), so concerns over the entailed philosophical commitments of “constitution” terminology should not be reason to avoid such a construct, or something like it^5^. Moreover, once we admit talk of such notions, questions arise regarding: (i) whether by minimally sufficient NCrC, we mean the very same thing as the neural basis, mechanism, substrate, or constitution of consciousness; (ii) whether these constructs pick out the same or different neural activity sets; and (iii) if they could pick out *different* neural activity sets, which should be considered the ultimate empirical target for consciousness science.

## STEPWISE INHIBITION

The notion of the minimally sufficient NCrC was created to distinguish it from the *merely* sufficient NCrC (with a *necessity* criterion being considered altogether too strong; Chalmers, 2000). However, the distinction between the merely and minimally sufficient NCrC *entails* an empirical strategy. If we are to arrive at identification of the minimally sufficient NCrC, we will presumably need to *remove* NCrCs one by one to assess whether each has minimally sufficient status. It is critical here to note that when I refer to an NCrC being minimally sufficient, or having minimally sufficient status, I mean to say that it is a *part* of the whole minimally sufficient neural activity set. Similarly, when I refer to an NCrC being constitutive, or having constitutive status, I mean to say that it is a *part* of the whole NCnC. Returning to the empirical approach to reducing the merely sufficient NCrC to the minimally sufficient NCrC, the most obvious means of doing this is by *stepwise inhibition* (disablement) of neurons, neuron types^6^, local or distributed neural circuits, specific brain regions or sets of NCrCs.

Chalmers’ definitions would seem to imply something like a stepwise inhibition empirical strategy when he comments (2000, p. 25), “In this way, we pare down any potential NCC to its core: Any irrelevant material will be whittled away, and an NCC will be required to contain only the core processes that suffice for the conscious state in question.” There is some conflict in Chalmers’ view here, given his willingness to whittle away but not to lesion. We therefore need to qualify the *type* of inhibition to be employed in identifying the minimally sufficient NCrC, and select methods that do not induce gross lesions and consequent architectural disruption (such as that induced by stroke, tumor, injury, or surgery). Although current methods such as inhibitory transcranial magnetic stimulation could be employed—and indeed this method provides striking and immediate perceptual disruption during binocular rivalry (Miller et al., 2000; see also Ngo et al., 2013)—for conceptual clarity we might postulate future highly specific molecular knockout techniques in which this or that NCrC can be selectively and reversibly inhibited. Such techniques were predicted by Crick and Koch (1998; see also the quote further below from Fenno et al., 2011) and are *currently* being developed and refined, with stunning progress, in the field of optogenetics (as detailed further below; Fenno et al., 2011; see also Klink et al., 2013). Although harmless application of such techniques in humans remains a very long way off, we can nonetheless begin to think through how such highly selective inhibitory techniques could, *in principle*, assist with identification of the NCnC.

As I have previously noted (Miller, 2001, 2007), if we inhibit an NCrC and consciousness disappears (or degrades), this might suggest the inhibited NCrC *is* constitutive in the normal case but does not actually prove its constitutive status. This is because such an NCrC might simply be *necessary and supportive* for consciousness without being constitutive of it. Conversely, if we inhibit an NCrC and consciousness does not disappear (or degrade), this might suggest the inhibited NCrC is *non*-constitutive in the normal case but does not prove such non-constitutive status. This is because such an NCrC might simply be *redundantly* constitutive. Because of these uncertainties, I have claimed that stepwise inhibition does not lead to conclusions regarding the NCnC. However, it certainly *does* lead to conclusions regarding the minimally sufficient NCrC.

## THE JENGA ANALOGY

**FIGURE 1:**
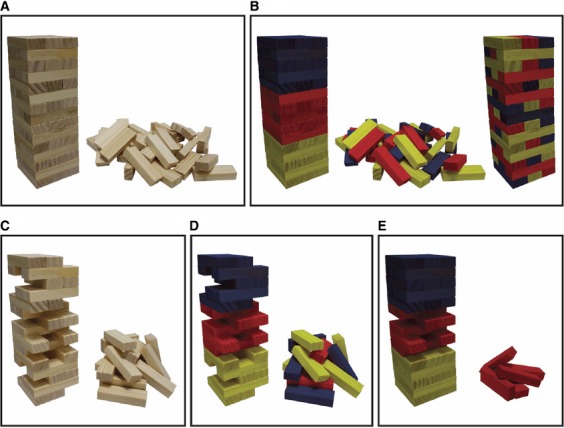
**The Jenga analogy schematizes the stepwise inhibition strategy inherent in distinguishing the merely sufficient from the minimally sufficient NCrC and shows how the minimally sufficient NCrC can differ from the NCnC.** In all panels, the upright tower represents the presence of a specific conscious state (or content) while in panels (**A,B**) the fallen tower (rubble) represents the loss (or degradation) of that state. Each block represents a perception-dependent NCrC that correlates with the specific conscious state (i.e., a specific factor NCrC). The whole tower is what remains after a process of screening off non-correlated neural activities and NCrCs that are only loosely correlated with consciousness or whose precise timing or known mechanistic function indicates they are not candidates for the NCnC. Panels **(A,C)** depict real-world situations, while panels (**B,D,E**) depict colored subdivisions that could only be identified if the Cr/Cn distinction problem is solvable. In (**B,D,E**), yellow blocks represent tightly correlated non-constitutive upstream activities (NCrC precursors/prerequisites), blue blocks represent tightly correlated non-constitutive downstream activities (NCrC consequences) and red blocks represent correlated constitutive activities (the NCnC). The right tower in **(B)** represents a more distributed depiction of the colored subdivisions than the left tower in **(B)**. In **(C,D)**, the upright tower with NCrC blocks removed is at the critical point beyond which any further block removal will result in the disappearance (or degradation) of the conscious state and collapse of the tower. The upright tower in **(C,D)** thus represents the minimally sufficient NCrC. Blocks in this critical point tower have minimally sufficient status, while those removed from it do not. In P1 (see main text), the possibility of non-constitutive minimally sufficient NCrCs (remaining yellow and blue blocks in **D**) means that the minimally sufficient NCrC could be a larger set of neural activities than the neural basis, mechanism, substrate, or constitution of consciousness (i.e., the tower in **D** is a larger set of blocks than the red middle third of the left tower in **B**). In P2 (see main text), the possibility of redundancy in the NCnC (i.e., the possibility of constitutive non-minimally sufficient NCrCs; removed red blocks in **E**) means that the minimally sufficient NCrC could be a smaller set of neural activities than the neural basis, mechanism, substrate, or constitution of consciousness (i.e., the red remaining blocks in the tower in **E** is a smaller set of blocks than the red middle third of the left tower in **B**; note P1 and P2 are not meant to be considered together—see main text). The stepwise inhibition strategy can therefore be used to assign minimally sufficient status to each block in the Jenga tower in **(A)** (thus the real-world situation of getting from **A** to **C**). However, the Cr/Cn distinction problem claims that there are no evident strategies to readily assign color status (constitutive red versus non-constitutive yellow/blue) to each of the NCrC blocks (thus it is not clear how to get from **A** to **B**). The analogy shows that although we might get from **(A)** to **(C)** using the stepwise inhibition approach, this will not satisfy consciousness science because we still cannot assign color status to either the remaining or removed blocks in **(C)** (we cannot get from **C** to **D**). Through this analogy it is claimed that the ultimate target of consciousness science is not to identify the minimally sufficient NCrC, but rather to distinguish constitutive (red) NCrCs from non-constitutive (yellow/blue) NCrCs. That is, it is ultimately the neural basis, mechanism, substrate, or constitution of consciousness that we seek to understand and this construct can pick out a different neural activity set from that of the minimally sufficient NCrC. Figure and caption reprinted with permission from Miller (in press-b).

To illustrate how the minimally sufficient NCrC and the NCnC are constructs that can pick out different neural activity sets, and to schematize the stepwise inhibition approach described above, I have proposed the Jenga analogy (Miller, in press-b). This analogy is from the popular game in which blocks are removed from a tower structure, one by one, until eventually a *critical point* is reached and the tower falls. An upright tower in this analogy (Figure 1) represents a *specific* conscious state or content being present while the fallen tower represents the absence (or degradation) of that conscious state or content. Each block in the tower represents an NCrC that correlates with that specific conscious state or content. This follows the distinction by Koch (2004) between “specific factors” and “enabling factors,” with the former dealing with particular conscious states or content and the latter with the overall state of being conscious. Although this distinction is itself the subject of considerable conceptual and methodological controversy (see Noë and Thompson, 2004; Bayne, 2007; Hohwy, 2007, 2009; Neisser, 2012; Bayne and Hohwy, 2013; Hohwy and Bayne, in press), I sidestep this debate and constrain the ensuing discussion to just specific factors^7^.

**FIGURE 2:**
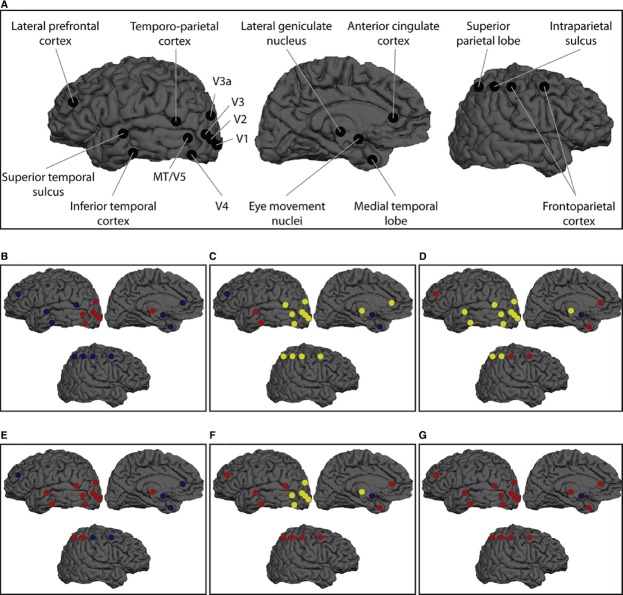
**The Cr/Cn distinction problem is clearly illustrated with reference to the phenomenon of binocular rivalry. (A)** Research using electrophysiological (single-unit and local field potential), brain-imaging and brain stimulation (and inhibition) approaches has identified a wide array of NCrCs during binocular rivalry and related phenomena such as flash suppression (for details, see Miller, 2013a, in press-b; for even further electrophysiological NCrCs during rivalry and related phenomena, see Figure 6 in Boly et al., 2013). The existence of multiple NCrCs during rivalry raises the issue of which activities can be said to constitute a specific conscious state rather than being merely correlated with it. There are multiple hypotheses possible for which NCrCs are upstream from (i.e., precursors or prerequisites), which are downstream from (i.e., consequences), and which are constitutive of, consciousness. These competing hypotheses are indicated by the color-coding alternatives in the smaller panels (**B–G**, which follow the color coding of the Jenga analogy in Figure 1). While this NCrC array could be subjected to the stepwise inhibition strategy underlying the notion of the minimally sufficient NCrC, the problem of assigning the appropriate color-coding to each NCrC cannot be solved with the same strategy because of the Cr/Cn distinction problem. There are several caveats to interpreting this figure, including issues such as: not all neurons in each area will exhibit perception-dependent firing (e.g., V1/V2, V4, middle temporal area, MT); even when perception-dependent in V4 and MT, this includes a proportion of neurons with the opposite expected firing pattern (i.e., lower firing rates when their preferred stimulus is perceived and higher firing rates when their preferred stimulus is suppressed); some of the regions correlate with transitions between rivaling states (or with reporting of such states) rather than with the visual states themselves; some regions are targets of rivalry temporal parameter modulation rather than consciousness modulation per se; regions engaged in attentional selection and top-down modulation could be considered either upstream or downstream activities; and there may be important binding mechanisms that physiologically link individual NCrCs (see main text). Figure reprinted and caption adapted with permission from Miller (in press-b).

As the case of binocular rivalry illustrates (Figure 2A; see Miller, 2013a, in press-b), there are many (specific factor) NCrCs for a given conscious state and these can include specific brain regions or specific neural populations within brain regions. In the Jenga analogy, each NCrC block can thus be considered representative of a specific factor NCrC at either local, distributed or regional levels^8^. The first step in the stepwise inhibition strategy is to use recording techniques to create an NCrC specific factor map for a specific (target) conscious state. Several further stipulations for the Jenga analogy are required. First, the entire tower is considered to be the outcome of previous strategies to “screen off” irrelevant *neural* activities (Hohwy, 2009; Hohwy and Bayne, in press). Hence, on this analogy, already screened off would be (i) neural activities that do *not* correlate with specific conscious states or content; (ii) neural activities that correlate with specific conscious states or content, but do so only in a *loose* fashion, in which the correlation can be broken one way or another^9^; (iii) neural activities that correlate with specific conscious states or content but whose precise *timing* provides clear ascription of either upstream or downstream, rather than constitutive, status (see Miller, in press-b for details; see also Aru et al., in press; de Graaf and Sack, in press; Hohwy and Bayne, in press; Revonsuo, in press)^10^; and (iv) neural activities that correlate with specific conscious states or content but whose known mechanistic functions provide grounds for clear ascription as non-constitutive^11^. What remains after such a screening off process therefore, is a tower of neural activities that *tightly* correlate with a specific conscious state or content and whose precise timing or known mechanistic functions cannot be used to accurately ascribe upstream, downstream, or constitutive status^12^.

Second, NCrC blocks in the lower third of the tower (colored yellow) represent tightly correlated non-constitutive upstream activities (tightly correlated NCrC precursors or NCrC prerequisites), those in the upper third (colored blue) represent tightly correlated non-constitutive downstream activities (tightly correlated NCrC consequences), and those in the middle third (colored red) represent tightly correlated *constitutive* NCrCs (the NCnC). Third, the upright tower at the critical point beyond which any further block removal will result in its collapse and the disappearance (or degradation) of the target conscious state represents the minimally sufficient NCrC.

Consider now that each of the thus-defined tightly correlated neural activity blocks in the Jenga tower can be described by only *one* of the following conjunctions:

(i) non-constitutive and non-minimally sufficient
(ii) non-constitutive and minimally sufficient
(iii) constitutive and non-minimally sufficient
(iv) constitutive and minimally sufficient

Consider further, the following two possibilities (P), each of which is to be considered in isolation (because complexities arise when they are considered together):

Possibility 1 (P1): Considering all individual NCrCs that have survived the screening off process, the possibility of non-constitutive minimally sufficient NCrCs means that the minimally sufficient NCrC could be a *larger* set of neural activities than the neural basis, mechanism, substrate, or constitution of consciousness.

Possibility 2 (P2): Imagining just the neural basis, mechanism, substrate, or constitution of consciousness, the possibility of redundancy (the possibility of constitutive non-minimally sufficient NCrCs) means that the minimally sufficient NCrC could be a *smaller* set of neural activities than the neural basis, mechanism, substrate, or constitution of consciousness.

Suppose now, that the first five correlated non-constitutive upstream activity blocks are removed but the conscious state remains and the tower stays upright. Those NCrCs are therefore *non*-minimally sufficient. Further suppose, however, that removal of the sixth correlated non-constitutive upstream activity block does lead to the disappearance (or degradation) of the conscious state and to falling of the tower. That sixth NCrC *is* minimally sufficient. But the Jenga analogy shows that an NCrC can be minimally sufficient *without* necessarily being constitutive. In this way, the minimally sufficient NCrC could be a larger set of neural activities than the NCnC (Figure 1). The downstream case is a little more complex (see Aru et al., in press; de Graaf and Sack, in press; Hohwy and Bayne, in press; van Boxtel and Tsuchiya, in press), and it is not always clear what should be regarded as an upstream activity and what a downstream activity, particularly given the unclear neurophysiological role of feedback. However, the difference between upstream and downstream cases is not particularly important for the point I am making and P1 is illustrated clearly with reference to just the upstream case.

Next imagine the set of neural activities that is the NCnC and consider that due to the possibility of redundancy in this neural activity set, five NCrC blocks could be removed without the disappearance (or degradation) of the conscious state and with the tower remaining upright. Those five neural activities are therefore *non*-minimally sufficient despite being actually constitutive. Removal of the sixth correlated constitutive neural activity block, however, takes the tower passed its critical point and the conscious state disappears (or degrades) and the tower falls. This sixth NCrC block then is *both* constitutive and minimally sufficient. In this way, the minimally sufficient NCrC within this (imagined) constitutive neural activity set could be a smaller set of neural activities than the NCnC (Figure 1).

The issue of claiming larger versus smaller sets is complicated when conceiving of P1 and P2 *together*, but the key message here is not about the overall size of the different neural activity sets, but rather that the minimally sufficient NCrC and the NCnC *can be different sets of neural activities*. Other important complexities include combinatorial and order complexities (i.e., whether the critical block removal would be critical *whenever* it is removed, or only when removed *after* removal of the previous five blocks, or only after removal of the previous five blocks in *that* specific order). Despite these complexities, what is important here is that while the empirical strategy of stepwise inhibition can identify an NCrC’s minimally sufficient status, it cannot identify its constitutive status. As the Jenga analogy shows, and as depicted in Figure 2 using the case of binocular rivalry, while stepwise inhibition achieves identification of the minimally sufficient NCrC, it cannot identify (i) which of the *remaining* blocks are constitutive of the conscious state and which are not; or (ii) which of the *removed* blocks are constitutive of the conscious state and which are not^13^.

This is not to say that identifying the minimally sufficient NCrC through stepwise inhibition would be an insignificant achievement for consciousness science. On the contrary, it would be a *major* achievement. However, the problem remains of just how science will experimentally distinguish the neural activity sets of the minimally sufficient NCrC and the NCnC (or if these are in fact the very same sets, how this can be shown to be the case). In my view then, the minimally sufficient NCrC construct should not be considered the ultimate empirical target for consciousness science because it could *include* neural activities that are *not* part of the neural basis, mechanism, substrate, or constitution of consciousness (P1) and it could *exclude* neural activities that *are* (P2). The minimally sufficient NCrC notion, for all its worth, subtly shifts the target of consciousness science to an empirically tractable one, while the real target remains elusive due to the Cr/Cn distinction problem.

## STEPWISE STIMULATION AND COMBINED INHIBITION/STIMULATION

If recording strategies make evident the Cr/Cn distinction problem and stepwise inhibition can identify an NCrC’s minimally sufficient but not constitutive status, what might the strategy of neural stimulation achieve? And indeed, exactly how would such a strategy be applied in the context of studying consciousness? Perhaps the first distinction required here is that between stimulation of currently *inactive* NCrCs and those *already active*. On the one hand, stimulation of already active correlated neurons, neuron types, local or distributed neural circuits, brain regions or sets of NCrCs, would generally not be expected to change a conscious state (irrespective of whether these stimulated NCrCs are constitutive or not). On the other hand, when stimulating currently *inactive* correlated neurons, neuron types, local or distributed neural circuits, brain regions or sets of NCrCs, a changed conscious state might suggest a constitutive role for such NCrCs but such a role is not proven by this strategy. This is because the stimulated NCrC might in fact be *non*-constitutive with its stimulation simply activating downstream NCrCs that *are* constitutive. In this case there may be conclusions possible regarding the *causal chain* of neural processing for that state, but there are no corollaries regarding the NCnC^14^.

What about *combinations* of inhibition and stimulation? I have previously asserted (Miller, 2007, p. 165) that, “by recording from, disabling and stimulating various NCrCs, there do not seem to be any obvious corollaries regarding the NCnC.” However, development of the Jenga analogy now leads me to reassess this assertion. Consider the following experimental scenario—the *reverse* Jenga strategy—which might at least *in principle*, and *partially*, address issues of constitution. This strategy again requires a highly specific and powerful inhibitory, and now also stimulatory, technical capacity, i.e., the ability to selectively inhibit, disinhibit, and stimulate specifically tagged neurons, neuron-types, local or distributed neural circuits, specific brain regions and sets of NCrCs. Note here also that stimulation is a *further* physiological step beyond mere disinhibition to resting state activity. It is also to be noted that the arguments above and below concern inhibition and stimulation of *excitatory* rather than inhibitory neurons, though it is acknowledged that (i) physiological inhibitory neural activity is a fundamental feature of cortical microcircuits and is “electrically inseparable from excitation” (Borchers et al., 2012, p. 66); (ii) neurons that correlate with a specific conscious state by *decreasing* their firing rate are not addressed by these arguments; and (iii) *modulation* of neural activity is not addressed by these arguments. All of these issues (and those in footnote 13) make the arguments I wish to present more complex than is required at this stage, but I do not discount their relevance.

Again optogenetics comes to mind when positing a highly specific and powerful inhibitory and stimulatory technique and it is worth quoting in full, the first two paragraphs of a recent review of this technique (Fenno et al., 2011, p. 390, square brackets in original):

In describing unrealized prerequisites for assembling a general theory of mind, Francis Crick observed that the ability to manipulate individual components of the brain would be needed, requiring “a method by which all neurons of just one type could be inactivated, leaving the others more or less unaltered” (Crick 1979, p. 222). Extracellular electrical manipulation does not readily achieve true inactivation, and even electrical excitation, while allowing for temporal precision in stimulating within a given volume, lacks specificity for cell type. However, pharmacological and genetic manipulations can be specific to cells with certain expression profiles (in the best case) but lack temporal precision on the timescale of neural coding and signaling.

Because no prior technique has achieved both high-temporal and cellular precision within intact mammalian neural tissue, there has been strong pressure to develop a new class of technology. As a result of these efforts, neurons now may be controlled with optogenetics for fast, specific excitation or inhibition within systems as complex as freely moving mammals [for example, with microbial opsin methods, light-induced inward cation currents may be used to depolarize the neuronal membrane and positively modulate firing of action potentials, while optical pumping of chloride ions can induce outwards currents and membrane hyperpolarization, thereby inhibiting spiking (Figure 1)]. These optogenetic tools of microbial origin (Figure 1) may be readily targeted to subpopulations of neurons within heterogeneous tissue and function on a temporal scale commensurate with physiological rates of spiking or critical moments in behavioral tests, with fast deactivation upon cessation of light. With these properties, microbe-derived optogenetic tools fulfill the criterion set forth by Crick in 1979 (Deisseroth 2010, 2011).

Others have commented on the prospects for consciousness science offered by optogenetics (e.g., Tononi and Koch, 2008)^15^. The technique has recently been applied in mice to examine top-down modulation of visual processing (Zhang et al., 2014) and can be applied in *Drosophila* in the context of visual rivalry (Miller et al., 2012). For examples of brain stimulation techniques currently applicable in humans, including those already applied or capable of being applied to binocular rivalry, such as transcranial magnetic stimulation, vestibular stimulation techniques, transcranial direct current stimulation, and electrical microstimulation, see Been et al. (2007), Borchers et al. (2012), Cohen and Newsome (2004), Histed et al. (2013), Klink et al. (2013), Law et al. (2013), Ngo et al. (2013), Reppas and Newsome (2007), Sengpiel (2013), Sterzer (2013), and Thomson and Fitzgerald (2013). Despite the value of such techniques for stimulating (and in some cases inhibiting) neural activity, they entail various disadvantages and interpretive complexities such as: (i) whether they in fact cause stimulation or inhibition; (ii) their spatial imprecision and hence unintended effects on other local and regional neural targets; (iii) individual variation, regional variation, and neuronal morphological variation in stimulatory and inhibitory thresholds (and consequent perceptual and behavioral effects); and (iv) the ability of such techniques to be detected by the subject. The in principle “pure” inhibition/stimulation methodology on which the arguments in the present paper are grounded would avoid these problems, as far as is physiologically possible. And of course, knowing just how far this is physiologically possible will require a great deal of further neurophysiological understanding (see, for example, the interpretive cautions outlined by Logothetis, 2010, regarding emerging optogenetic studies).

Nonetheless, with an optogenetics-style technique as an example of the type of in principle methodology to which I am referring, consider that the first step in the reverse Jenga scenario is to use recording techniques to create a tightly correlated NCrC specific factor map for a specific (target) conscious state^16^. Next, leaving enabling factor NCrCs untouched, *all* previously mapped specific factor NCrCs are inhibited^17^. In addition, all non-correlated causal chain components, and all loosely correlated NCrCs, are inhibited. The crucial final intervention then is to stepwise disinhibit *and* activate (stimulate) each previously mapped tightly correlated specific factor NCrC. Under these circumstances, if the target conscious state is reported^18^ then this would seem to provide the strongest evidence possible that the disinhibited and stimulated NCrC is actually constitutive (and thus has constitutive status).

Note that in the case of P1 with the stepwise inhibition strategy, the reason a non-constitutive but minimally sufficient NCrC is minimally sufficient is because of its *input* to the NCnC (i.e., its role in causal chain processing, albeit in this case, a correlated rather than non-correlated causal chain role). Without such minimally sufficient non-constitutive NCrC activity, there could not be the required activity in the NCnC and there could not thus be the conscious state. However, in the reverse Jenga case just described, the NCnC is *directly* activated (stimulated) and thus, unlike the stepwise inhibition case, there is no dependency on minimally sufficient non-constitutive NCrCs. So in the reverse Jenga case, with this dependency condition removed, if the target conscious state appears with particular NCrC stimulation—and with other NCrCs inhibited, given the strategy is a *stepwise* combined inhibition/stimulation process—this reasonably implies constitutive status of the stimulated NCrC.

**FIGURE 3:**
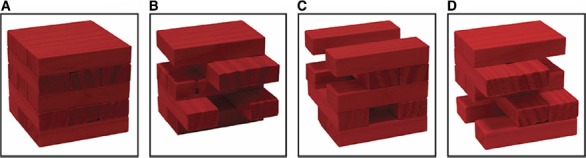
**The Jenga analogy illustrates notions of redundancy in the NCnC and non-radical neural multiple realizability.** The isolated stimulation strategy is (arguably) able to identify at least some constitutive neural activities (red colored blocks), specifically the minimally sufficient NCnC **(B)**, though it is not able to identify the boundaries of the whole NCnC **(A)**. Hence while **(B)** may be identified using isolated stimulation, we are not able to get from **(B)** to **(A)** with this method. Moreover, the isolated stimulation strategy could demonstrate that more than one subset of constitutive neural activities is minimally sufficient constitutive neural activity (e.g., **C** and **D**) and if so, **(B)**, **(C)**, and **(D)** would be non-radical neural multiple realizations of a specific conscious state. Figure and caption reprinted with permission from Miller (in press-b).

With the reverse Jenga strategy, we are thus able to *build* the middle third of the tower to its critical point of minimal sufficiency. Indeed, by identifying the tower at this critical point of just its middle third, it can be stated that what has in fact been identified is the *minimally sufficient NCnC*^19^. The reverse Jenga approach thus, arguably (see later), provides *partial* solution to the Cr/Cn distinction problem because it overcomes the obstacle of P1 and identifies *some* constitutive neural activities. However, to fully solve the Cr/Cn distinction problem, we need to achieve identification of *the whole set* of constitutive neural activities. To do that would require also overcoming the obstacle of P2 which would involve identifying not just the middle third of the tower at its critical point of minimal sufficiency, but the exact *boundaries* of that (whole) middle third (i.e., the exact boundaries of the NCnC; Figure 3).

To show why the reverse Jenga approach fails to fully solve the Cr/Cn distinction problem, consider the following further specific combined inhibition/stimulation scenarios (herewith I refer to the combined inhibition/stimulation approach as “*isolated* stimulation”). First, isolated stimulation of non-constitutive NCrCs in the bottom or top third of the tower—whether they are minimally sufficient NCrCs or not—would *not* induce the target conscious state. Consequently, these NCrCs can be reasonably excluded as constitutive neural activities. Second, after the conscious state first appears due to isolated stimulation of the minimally sufficient NCnC, and then as redundantly constitutive NCrCs are *additionally* disinhibited and stimulated, the conscious state will *not* change, so this will not allow a distinction to be made between redundantly constitutive NCrCs and non-constitutive NCrCs. Hence the whole set of constitutive neural activities cannot be identified.

The next isolated stimulation scenario is also informative. Thus, we can ask what would occur with isolated stimulation of redundantly constitutive NCrCs when the minimally sufficient NCnC is not also stimulated (i.e., whether that would induce the conscious state). Here the issue depends on the nature of the redundancy and the notion of *neural multiple realizability* (see Miller, 2007). That is, it may be that the minimally sufficient NCnC is a *fixed* set of neural activities for a particular conscious state, without activation of which there will never be that conscious state. In such a scenario—which invokes the notion of *necessity* rather than sufficiency—the usual case would involve a conscious state being constituted by activation of that fixed minimally sufficient neural set *and* by any additional redundantly constitutive neural activities. But another alternative is that the minimally sufficient NCnC may be a *variable* set of neural activities for a particular conscious state, such that *separate* isolated stimulation of two or more different sets of neural activities could induce (and constitute) the target conscious state, even though in the normal case *both* or *all* of these sets are constitutive. This would be a case of neural multiple realizability, whereby two or more different neural states could nonetheless constitute the same phenomenal state^20^. Note that this notion of neural multiple realizability, however, is still one in which redundancy is involved.

There is yet another, perhaps extreme, alternative in which neural multiple realizability could occur without any involvement of redundancy. That is, it could be that the whole scenario of isolated stimulation radically changes the NCnC, such that neural activities that are never constitutive in the normal case (perhaps tightly correlated upstream or downstream NCrCs) *become constitutive* in the case of isolated stimulation. We might describe this as *radical* neural multiple realizability, in which isolated stimulation of non-constitutive NCrCs in the bottom or top third of the tower—whether they are minimally sufficient or not—*could* conceivably induce (and constitute) the target conscious state. If this extreme scenario were to hold, it would mean that constitutive neural activities would still be identified by the isolated stimulation strategy but that such constitutive activity would bear no relationship to constitutive activity in the normal case. Achieving that identification would be far less relevant to consciousness science than achieving identification of constitutive activity in the normal case.

While radical neural multiple realizability cannot be totally excluded as a possibility, its likelihood can be questioned. That is, while the brain exhibits remarkable capacity for rapid and substantial plastic change—such as reorganization of somatosensory maps following deafferentation (Merzenich et al., 1983; Ramachandran et al., 1992; Weiss et al., 2000)—such changes nonetheless take days to weeks to occur. While radical neural multiple realizability for a specific conscious state may be highly probable after days to weeks, for it to confound the isolated stimulation strategy, it would need to involve more or less instantaneous reorganization of the NCnC. Instantaneous reorganization of the NCnC in this way would seem highly *im*probable.

Non-radical neural multiple realizability (Figure 3), however, is a far more likely possibility, as is redundancy in the NCnC, certainly in the case of within-region neural activity (see next section and footnote 20). Both non-radical neural multiple realizability and redundancy, given their higher probability, challenge consciousness science and account for the isolated stimulation strategy’s failure to identify the exact boundaries of the NCnC. To reiterate what each possibility involves, recall P2 above in the case of redundancy, and in the case of non-radical neural multiple realizability, we can state:

Possibility 3 (P3): Non-radical neural multiple realizability involves the possibility that more than one *subset* of constitutive neural activity can be minimally sufficient for a conscious state when separately subjected to isolated stimulation.

In summary then, with the isolated stimulation empirical strategy, while the obstacle of P1 is overcome (arguably, see later), the obstacles of P2 and P3 are not. And while minimally sufficient constitutive neural activities might be identified by isolated stimulation, redundantly constitutive neural activities cannot be distinguished from non-constitutive neural activities (P2) and hence the whole NCnC cannot be identified. Moreover, initially identified minimally sufficient constitutive neural activities may turn out to not be the *only* minimally sufficient constitutive neural activities (P3). Nonetheless, the reverse Jenga scenario may enable identification of some constitutive neural activities (indeed, minimally sufficient constitutive neural activities) and such identification would represent very significant progress in consciousness science. However, because this strategy fails to identify the exact boundaries of the NCnC, the Cr/Cn distinction problem is not solved and we may therefore only *close in* on the constitution of consciousness^21^,^22^.

## CLARIFICATIONS

At this stage, some points of clarification are required before proceeding to discussing potential objections to the presented arguments, and then to proposing new foundational claims for consciousness science.

### BETWEEN- AND WITHIN-REGION CASES

When considering the notions of redundancy in the NCnC (P2) and non-radical neural multiple realizability (P3), it should be noted that these possibilities apply both *between* and *within* specific brain regions. In Figure 2, the stepwise inhibition strategy and the obstacles of P1 and P2 were illustrated for the case of binocular rivalry. This phenomenon also serves to illustrate P2 and P3 in the between- and within-region cases. Thus, constraining our analysis to perception-dependent neural activities (which the Jenga and reverse Jenga analogies require), and setting aside the fact that rivalry is not normal vision, consider the following.

In the between-region case, a visual state (during rivalry, with motion, color and complex images) is constituted say, by IT/STS (inferior temporal/superior temporal sulcus) neural activity *and* V4/MT (middle temporal) neural activity (this being the whole specific factor NCnC for each rivaling state), but (i) the visual state would not be affected by inhibition of V4/MT activity because V4/MT activity is redundantly constitutive; *and* (ii) isolated stimulation of IT/STS neurons induces the visual state and is thus minimally sufficient constitutive neural activity for it, but isolated stimulation of V4/MT activity does not induce the visual state and thus is not minimally sufficient constitutive neural activity for it; *or* (iii) separate isolated stimulation of either IT/STS or V4/MT induces the visual state and thus both can be considered minimally sufficient constitutive neural activity for it (and non-radical multiple realizations of it).

In the within-region case, a visual state during rivalry is constituted say, by IT/STS neural activity (this being the whole specific factor NCnC for each rivaling state), but (i) the visual state would not be affected by inhibition of *some* IT/STS neural activity because that inhibited activity is redundantly constitutive; *and* (ii) isolated stimulation of some IT/STS activity induces the visual state and is thus minimally sufficient constitutive neural activity for it, but isolated stimulation of the remaining (or some other) IT/STS activity does not induce the visual state and is thus not minimally sufficient constitutive neural activity for it; *or* (iii) separate isolated stimulation of either some IT/STS activity or of remaining (or some other) IT/STS activity induces the visual state and thus both can be considered minimally sufficient constitutive neural activity for it (and non-radical multiple realizations of it).

While the between-region case above is certainly conceivable, the within-region case, especially for the redundancy possibility (P2), must be considered *highly probable*. That is, it is highly probable that thousands, tens of thousands, hundreds of thousands or millions of neurons are specific factor constituents for a conscious state, and it is highly improbable that every one of them would need to be active to constitute that state. Hence it is highly improbable that every one of them would need to be stimulated in the isolated stimulation case, to induce (and constitute) the target state. Within-region redundancy also raises further issues of importance for consciousness science and I address these later when considering an objection to the redundancy argument.

It is important to also be mindful in this context, however, of existing data from electrical microstimulation studies. It is a remarkable fact from such studies that stimulation of relatively few MT neurons (perhaps just hundreds) can *bias* a monkey’s perceptual decision regarding motion direction (Britten et al., 1992; Salzman and Newsome, 1994; Ditterich et al., 2003; Cohen and Newsome, 2004, 2009; Shadlen and Kiani, 2013; see Sengpiel, 2013, for discussion of this data in the context of binocular rivalry and for the point that microstimulation has never been applied to rivalry). One cannot necessarily extrapolate from those studies, however, in which stimulation of relatively few neurons can influence discrimination under difficult decision-making conditions, to the notion that there will be similarly few neurons constitutive for a specific conscious state. That is, it does not follow that the number of neurons it takes to bias competition within pools of active neurons, and to thus influence a subject’s difficult decision, is equivalent to the number of specific factor neurons constituting a conscious state. There may be similarly few neurons determining competition between conscious states (such as during rivalry) but this is a different matter to the number of specific factor neurons constituting each state.

Nonetheless, it may turn out that the activity of fewer neurons than we expect is the (specific factor) neural basis, mechanism, substrate, or constitution of a conscious state. This will, at the end of the day, be an empirical matter that strategies like isolated stimulation, should they come to pass, will determine. It is also noteworthy that Newsome himself is not afraid to examine the issue of just what his and his contemporaries’ microstimulation work means for the issue of the neural basis, mechanism, substrate, or constitution of subjective experience (Cohen and Newsome, 2004; Reppas and Newsome, 2007). He concludes (Reppas and Newsome, 2007, p. 7) that, “The development of a non-invasive technique to modify precisely and locally neural activity in humans will probably be necessary to address such questions satisfactorily.”

### LINKING, BINDING, OR INDEX PROCESSES

I have previously acknowledged (Miller, 2007) that searching for this or that NCrC or set of NCrCs might be misguided and that it is instead a neurophysiological process *linking* or *binding* multiple NCrCs, such as, for example, recurrent processing (Lamme, 2006, 2010; see also Klink et al., in press) or oscillatory activity and temporal synchrony (Singer, 2001; Fries, 2005; Fries et al., 2005; though see Shadlen and Movshon, 1999; see also Sengpiel, 2013), that *indexes* the neural basis, mechanism, substrate, or constitution of consciousness. It is certainly possible that physiological processes between, or that bind, individual NCrCs could index phenomenally conscious neural activity over and above the tight correlations observed in each of those individual NCrCs. But if so, there will likely be various sets of recurrently, temporally or otherwise bound NCrCs for a conscious state, and we can ask which set should be assigned constitutive status and how will various hypotheses in this regard be tested. Perhaps each bound neural set could be considered an “individual” NCrC and the same obstacles of P1–P3 would apply.

Alternatively, even if there are not various sets of indexed or bound neural activities—because all such sets should rather be considered just one larger set—there can still be various hypotheses proposed regarding whether all individual NCrC components of that one larger set are constitutive, or just some. In other words, it can be claimed that a neurophysiological process linking or binding various individual NCrCs, indexes the neural basis, mechanism, substrate, or constitution of consciousness, but it is a *further claim* to hold that all neurons or all brain regions participating in such a process are constitutive. That further claim is itself a hypothesis, with rival hypotheses being based on the notion that only some neurons, neuron types, local or distributed neural circuits, specific brain regions or sets of NCrCs participating in the index/binding process are constitutive.

To test hypotheses in this regard would require intervening through stepwise inhibition and stepwise isolated stimulation to prevent and reintroduce, respectively, the relevant index or binding process, and observing what happens to the conscious state in each case. In such experiments, it may turn out that in the absence of the index or binding process there is *never* the conscious state and this would certainly inform consciousness science. But it may also turn out that inhibition of one component of an indexed/bound neural set does not lead to the absence of the index/binding process in remaining components of the set and does not lead to a change in the target conscious state. Would that inhibited component therefore be considered non-constitutive or redundantly constitutive? And could isolated stimulation of various subsets of indexed/bound NCrCs be minimally sufficient constitutive neural activity? The index/binding process case does not seem to enable us to avoid the obstacles of P1 and P2 for stepwise inhibition and P2 and P3 for stepwise isolated stimulation.

### A CAVEAT ON ISOLATED STIMULATION

The final point of clarification relates to earlier reference to isolated stimulation only *arguably* enabling identification of at least some constitutive neural activities. That is, the above discussion of possible index processes that link or bind different neural activities raises an important potential complication for the isolated stimulation strategy. A scenario could be postulated whereby isolated stimulation of the (putative) minimally sufficient NCnC could fail to induce the target conscious state if that neural activity set required for its correct functioning, intact connections to other (disinhibited and active) neural activity sets (such as non-correlated causal chain components, loosely correlated NCrCs or tightly correlated non-constitutive NCrCs). Such a scenario would potentially then lead to an inability to distinguish the minimally sufficient NCnC and non-constitutive minimally sufficient NCrCs (and even non-correlated causal chain components) and this is precisely the obstacle of P1. In such a scenario therefore, it could be claimed that isolated stimulation does *not* in fact overcome the obstacle of P1. This is a concern addressed further at the end of the next section, where I discuss a potential objection based on a postulated requirement for intact connection even to disinhibited and *inactive* neural sets.

## OBJECTIONS

There are at least six potential objections to the arguments presented thus far. Each of these is discussed in detail in Miller (in press-b). Here I list three of these and then discuss in detail only the three most relevant to the presented arguments. The objections not discussed here include: (i) *Definition objections*—that Chalmers’ (2000) definition of the minimally sufficient NCrC, including his discussion of redundancy technicalities, would exclude the sorts of scenarios I have discussed; (ii) *Specificity objection*—that the Cr/Cn distinction problem may not be specific to consciousness science, but rather applies in many scientific domains; and (iii) *Theoretical loading objection*—that the very notion of constitution is theoretically loaded (regarding the relation between mind and brain) in a way that the notion of correlation is not.

### TRIVIALITY OBJECTION

Another potential objection is that it could be claimed that redundancy (P2) and non-radical neural multiple realizability (P3) are but *trivial* possibilities. This objection would hold that if we had identified the minimally sufficient NCnC using the isolated stimulation strategy, we need not be concerned about failing to go on to achieve identification of the whole NCnC because any differences between these two neural activity sets is trivial. There are two responses to this objection and both draw on the notion of *explanation* in consciousness science (a notion discussed more fully in Chalmers, 1996; Revonsuo, 2000, 2001, 2006, in press; Hohwy and Frith, 2004; Bayne, 2007; Hohwy, 2007, 2009; Seth, 2009; Neisser, 2012; Drayson, in press; Hohwy and Bayne, in press; Keaton, in press; Miller, in press-b; Opie and O’Brien, in press).

Explanation is a somewhat vexed issue in consciousness science because of “the hard problem” discussed by Chalmers (1996) in which even identification of the whole NCnC could still leave unanswered questions such as *how* it is that *that* particular set of neural activities constitutes consciousness and *why* it is that there should be any consciousness at all. Despite the possibility of explanatory gaps in the study of consciousness (Levine, 1983), some degree of explanation *can* still be sought and achieved in the scientific study of consciousness, in particular with respect to the notion of *mechanistic explanation* in the biological sciences (Bechtel, 1994; Machamer et al., 2000; Revonsuo, 2000, 2001, 2006, in press; Craver, 2007; Horst, 2007; Neisser, 2012; Oizumi et al., 2014; Hohwy and Bayne, in press; Mahner, in press; Miller, in press-b; Opie and O’Brien, in press; cf. Irvine, 2013, who appeals to mechanistic explanation to argue why there cannot even be a science of consciousness)^23^.

It could be argued, as Jakob Hohwy has pointed out to me, that we need not worry about identifying redundantly constitutive neural activities because if a conscious state does not disappear (or degrade) when a redundantly constitutive NCrC is inhibited, then the difference between the minimally sufficient NCnC and the whole NCnC, is a *difference-without-a-difference* (and any such difference is therefore trivial). As such, the argument would hold that, being redundant, the unidentified constitutive NCrCs could do no *explanatory work* even if they were identifiable. My reply, however, is that a difference-without-a-difference for consciousness does not amount to a difference-without-a-difference for consciousness *science*.

That is, we can reasonably wish to answer the following questions: (i) is there in fact any redundancy in the NCnC? (ii) if so, why should there be such redundancy? (iii) is such redundancy based on a critical *size* of neural activities (a critical number of involved neurons, neuron types and neural circuits)? (iv) is such redundancy based on a critical *location* of neural activities? (v) is such redundancy based on a critical *combination* of stepwise inhibition when applying that empirical strategy? (vi) is such redundancy based on a critical *order* of stepwise inhibition when applying that empirical strategy? (vii) is there non-radical neural multiple realizability within the NCnC (which draws on the notion of redundancy) when applying the isolated stimulation empirical strategy? (viii) and how does such redundancy relate to index or binding processes? Answers to such questions would seem, in my view, far from devoid of explanatory power (just as understanding many such issues would help to *mechanistically explain* when and why a Jenga tower might fall).

The second response to the triviality objection rests on the fact that *the* major element of the scientific study of consciousness, at least currently, is the search not for explanations as such, but rather for identification of *which* neural activities are the basis, mechanism, substrate, or constitution of consciousness. This is fundamentally a process of identifying the relevant neural activities, not of explaining how and why *those* activities do the constituting. That is, the issue with which I have been concerned in this paper (and with which consciousness science appears most concerned) is one of determining inclusion and exclusion into the constitutive neural activity set. Understanding explanatory mechanisms may of course help with this identification process, but consciousness science could conceivably identify the NCnC even without understanding some explanatory mechanistic principles relevant to that neural activity set.

Hence in summary, appeals to the absence of explanatory power in redundantly constitutive NCrCs do not support their identification as trivial, because (i) issues regarding redundantly constitutive NCrCs can in fact do explanatory work; and (ii) despite their redundancy, redundantly constitutive NCrCs are nonetheless constitutive activities and are thus legitimately part of the neural basis, mechanism, substrate, or constitution of consciousness. As such, we should seek their identification and we should consider what scientific strategies might achieve this goal.

### WAIT-AND-SEE OBJECTION

A further potential objection concerns Crick and Koch’s (1998) suggestion that hard problems of consciousness be set aside until science makes more progress. It is not clear if such a caution should include the Cr/Cn distinction problem given its clear scientific relevance, but obviously in my view, it should not. That said, there is certainly an important message in the wait-and-see approach and indeed I have previously noted (Miller, 2007, p. 165) that, “… future scientific work may show that the notion of the Cr/Cn distinction is somehow fundamentally misguided (in a way that cannot yet be appreciated because the science is not yet done).” Similarly, Revonsuo (2006, p. 292) rightly cautions us over imaginary neuroscience scenarios when he says: “In the absence of the relevant empirical facts, we simply cannot imagine or foresee the perfect future science regarding *any* phenomenon.”

I am entirely accepting of the fact that as neuroscience progresses, particularly with respect to dynamic mechanistic multilevel explanation of neural processing within *and* outside of consciousness science, any or all of the scenarios and claims made in this paper (and its longer version) may require refinement, revision or indeed rejection. For example, the technique of optogenetics as applied in the context of the current paper would also be capable of being applied in many other neuroscientific contexts (e.g., sensory coding, voluntary movement and various executive functions). As such application advances understanding of neural coding, signaling, dynamics and function, and understanding of the relationship between neural processing and the phenomena being studied, so too such understanding will have flow-on implications for consciousness science. In addition to optogenetics, developments will occur (as discussed in several papers in Miller, 2013a) with other recording, inhibition, and stimulation methods, and signal analysis approaches therein, to improve the spatial and temporal resolution of each technique, to enable combined methodological approaches, to accurately map structural and functional neural connections, to improve neural spike detection and sorting and neural population, source and network modeling, to facilitate integration of knowledge across the various levels targeted by each technique, and in general to develop a more detailed understanding of subcellular, neural, neural circuit, and large-scale/regional/systems processing.

Conversely, as neuroscientific understanding progresses, the presented scenarios and claims may rather become strengthened (for example, the claim that the isolated stimulation strategy identifies some constitutive neural activities may not be questionable after all). Moreover, the presented scenarios and claims may go beyond the realm of “in principle” and instead become *directly testable*. Until there is development of a safe, reversible and “pure” recording, inhibitory and stimulatory technique, it would be unwise to not accept likely revision to the presented scenarios and claims. Just as this and other neuroscientific progress will help to disentangle the complexities of the microstimulation and decision-making literature, and the broader neural coding and processing issues within which those complexities lie (see references in previous section; reviewed in Shadlen and Kiani, 2013; see also Sengpiel, 2013), so too it will help to disentangle the complexities of recording, inhibition, and stimulation scenarios within consciousness science. We may wish to wait and see on such issues before getting too conceptually entangled, but in my view it would be unwise to hold that the conceptual analyses and proposals presented here should not be further discussed and debated, or should not even have been embarked upon.

### INTEGRATED INFORMATION THEORY OBJECTION

The final potential objection is that stemming from Integrated Information Theory (IIT; Oizumi et al., 2014; see also Klink et al., in press). IIT is a highly complex and developed theory of consciousness, with its own detailed conceptual definitions and tools, that starts with phenomenological axioms and proceeds to formalize these into, “postulates that prescribe how physical mechanisms, such as neurons or logic gates, must be configured to generate experience (phenomenology)” (Oizumi et al., 2014, p. 1). IIT is based heavily on mechanistic causal roles—though specifically in IIT, and importantly in the present context, only differences that make a difference—and involves perturbation (again important in the present context) of the elements of candidate sets into all possible states, and identification of maximally irreducible cause–effect repertoires and structure. IIT does not permit for P1, P2, or P3 because it takes the *inactive* elements in maximally irreducible cause–effect structure to be just as critical as the active elements. Additionally, through an exclusion postulate, among overlapping candidate sets of mechanistic elements, only one forms a complex—that with the maximum quantity of integrated conceptual information—and hence no subsets or supersets of those mechanistic elements can form a complex.

It will take someone better versed than myself in IIT to properly set out the detailed objections to P1, P2, and P3 that stem from the theory, but from the brief description above, some clues to the objections should be visible. The arguments presented in this paper are based on an excitation approach to constitutive neural activity, albeit with an acknowledgment of the physiological inseparability of excitatory and inhibitory processes (and other caveats). However, IIT considers inactive (i.e., resting state, or disinhibited and not stimulated) neural states to be just as critical to the constitution (structure) of a conscious state as stimulated neurons, and on this construal, the isolated stimulation scenario I have been discussing would be considered inaccurate. Note though, that this way of thinking would also lead to objecting to the notion of the minimally sufficient NCrC, insofar as that construct also relies on excitatory correlates rather than the set of relevant active *and* inactive neural elements.

Instead, IIT would argue that the constitution of consciousness is identifiable by identifying the (neural) complex with the maximum quantity of integrated conceptual information and that once this complex has been identified, adding anything to it or subtracting anything from it must change the conscious state, however minimal that change may be. On this construal and on the axioms and postulates of IIT, P1, P2, and P3 are not possibilities at all. This IIT objection to the present arguments is testable, in principle, and if the identified complex with the maximum quantity of integrated conceptual information can in fact be added to or subtracted from without a concomitant change in the conscious state, it will then be IIT that is found wanting.

## RELATED SCIENTIFIC AND PHILOSOPHIC ISSUES

There are many additional issues associated with the notion of constitution and the Cr/Cn distinction problem, with both explanatory and other theoretical implications (discussed in detail in Miller, 2007, in press-a,b). For example, additional scientific questions include: (i) what do the Cr/Cn distinction problem, the Jenga analogy and the reverse Jenga analogy look like for *enabling* rather than specific factors? (ii) what is the relationship of the Cr/Cn distinction problem to other scientific and philosophic consciousness problems—such as identifying where in phylogeny and where in ontogeny phenomenal consciousness exists, knowing what it is like to be another subject (the other minds problem or problem of direct intersubjective exchange), and the hard problem? (iii) if science meets an epistemic limit with the Cr/Cn distinction problem, what is it about scientific method that gives rise to such a limit?

Another important scientific issue concerns at what *level* of neural processing consciousness is constituted (Revonsuo, 2000, 2001, 2006, in press; Miller, 2007, in press-b; Hohwy and Bayne, in press; Opie and O’Brien, in press)? This is another constitution problem that both the science *and* philosophy of consciousness will need to address. Thus, should the NCnC be considered most relevant at the level only of constitutive action potentials? Or perhaps at the level only of the electrophysiological processes of distributed constitutive neural circuits and networks (bound as they may or may not be)? Or perhaps both levels are constitutively relevant? And what is the relation of NCnC *micro*constituents (subcellular molecular constituents) to consciousness? Should the NCnC be considered to include all microconstituent processes, or only some? And if just some, which, and how will we test hypotheses in this regard? In this context, the term “constitution” takes on additional relevance over and above “basis,” “mechanism” and “substrate” and specifically, different *grains* of constitution can be appreciated—i.e., at coarse (systems), fine (individual neurons and microcircuits), and very fine (subcellular) scales (see Miller, in press-b).

Some of the additional philosophic issues have been alluded to above in terms of other philosophic problems of consciousness. Also, the issue of various grains of constitution suggests, in my view, a *mereology of phenomenal consciousness*. Mereology is the branch of philosophy that deals with the relationship between parts and wholes. It has mostly concerned itself with analysis of part-whole relations for static objects (see several papers in Miller, in press-a) rather than *process*-based physiological systems. It is my contention that the philosophy of consciousness needs to (i) engage in mereological analysis of processes to better understand part-whole relations of physiological and specifically neural systems; and (ii) thereafter, focus on what additionally may be relevant in considering a mereology of the NCnC. A mereology of phenomenal consciousness will require concerted interdisciplinary interaction amongst philosophers and scientists, with updating of the analyses as neuroscientific knowledge itself progresses. Extension of the classical mereological focus on objects to (neurophysiological) processes should be undertaken to *complement* mechanistic explanation approaches (which also deal with component parts, and specifically their causal operations, activities, and organization)—particularly *dynamic* mechanistic explanation; see Bechtel and Abrahamsen, 2010, 2013)—rather than as an alternative to it^24^.

Other relevant philosophic issues surrounding the notion of constitution concern the perennial issue in philosophy of mind of the relation between brain and mind. Thus, (i) what are the similarities and differences between the constitution relation and others that purport to describe the brain–mind relation, such as identity, supervenience, realization, emergence, and causation? (ii) is the constitution relation in the case of phenomenal consciousness a unique constitution relation, and if so, unique how? (iii) if indeed unique, does it simply look nothing like a constitution relation in the usual parts-whole sense, even for processes? (iv) does the uniqueness of the constitution relation in this particular case tell us something about the uniqueness of consciousness and the place of consciousness in nature? Finally, another perennial issue in philosophy of mind—the ontological issue of whether consciousness and mind are entirely physically (materially) composed—is also relevant in the context of the Cr/Cn distinction problem. If with the Cr/Cn distinction problem, science meets an epistemic limit, what does this mean for these ontological matters? For more detailed discussion of these related scientific and philosophic issues, see Miller (2007, in press-a,b).

## NEW FOUNDATIONS FOR THE SCIENCE OF CONSCIOUSNESS

In this paper (and in Miller, in press-b), we have seen through the Jenga analogy that the minimally sufficient NCrC construct can pick out a different neural activity set to that of the NCnC. We have also seen that while application of the stepwise inhibition empirical strategy can distinguish the merely sufficient from the minimally sufficient NCrC, it fails to make any progress on identifying constitutive neural activities (due to P1 and P2). In addition, while application of the isolated stimulation empirical strategy can (arguably) identify some constitutive neural activities, it fails to make any progress on identifying the whole NCnC (due to P2 and P3).

Although the Cr/Cn distinction problem remains unsolved, as are other (mereological) consciousness constitution problems, the analyses I have presented suggest new conceptual foundations for consciousness science, depicted by the following claims (C). These claims are proposed for further discussion and notably, they include reference to specific empirical strategies and to possibilities P1–P3. There are many aspects of consciousness science not dealt with by these claims, so this is not intended to be an exhaustive list. It is merely a list of claims arising from the main analyses I have undertaken. The proposed claims are:

C1: Setting aside notions of the hard problem of consciousness, the explanatory gap, hard phylogeny and ontogeny problems of consciousness, and the problem of direct intersubjective exchange, the ultimate aim of consciousness science is not to identify the minimally sufficient neural correlates of consciousness, but rather to identify the (whole) neural basis, mechanism, substrate, or constitution of consciousness.

C2: The current foundational construct of consciousness—the minimally sufficient neural correlates of consciousness—can pick out a different neural activity set to that picked out by the neural basis, mechanism, substrate, or constitution of consciousness.

C3: If by “under conditions C” in Chalmers’ (2000) definition of the minimally sufficient neural correlates of consciousness, we mean stepwise inhibition—the most obvious empirical approach to distinguishing the merely sufficient from the minimally sufficient neural correlates of consciousness—then the minimally sufficient neural correlates of consciousness construct is limited by its inclusion of neural activities that are not in fact part of the neural basis, mechanism, substrate, or constitution of consciousness, and by its exclusion of neural activities that are. Consciousness science can nonetheless continue to work toward identifying the minimally sufficient neural correlates of consciousness using recording strategies and the stepwise inhibition strategy.

C4: If by “under conditions C” in the definition of the minimally sufficient neural correlates of consciousness we mean isolated stimulation, then the minimally sufficient neural correlates of consciousness construct is equivalent to the minimally sufficient neural constitution of consciousness construct and at least some constitutive neural activities will (arguably) be identifiable. Whichever term is preferred, this neural activity set can be different to that of the *whole* neural basis, mechanism, substrate, or constitution of consciousness and hence neither construct with the minimally sufficient qualifier should be considered the ultimate target of consciousness science.

C5: Because of the possibilities of redundancy in the neural constitution of consciousness (i.e., of constitutive non-minimally sufficient neural correlates of consciousness) and of non-radical neural multiple realizability, there is not yet evident an empirical strategy to identify the whole neural constitution of consciousness. Empirical approaches to this problem need to be developed, and may require entirely new scientific strategies^25^.

The science of consciousness is young and thriving. There is a great deal of empirical and conceptual work to be done in this field and the foundational map charted by Chalmers (2000) needs to be reassessed and built upon, with a focus on empirical strategies. The analyses and new foundational claims presented here (and in Miller, in press-b) are an attempt in this direction. Consciousness remains, as ever, an intriguing subject of intellectual discourse. It is today also rightly situated at the frontier of scientific endeavor.

### CONFLICT OF INTEREST STATEMENT

The author declares that the research was conducted in the absence of any commercial or financial relationships that could be construed as a potential conflict of interest.

## Footnotes

1 Note that the plural “NCrCs” indicates the many individual neural activities that correlate with a specific state of consciousness. This is different to “the NCrC” which is a collective term commonly used to denote the set of all NCrCs for such a state. More points of clarification and definition are detailed below.

2 I expect the problem to have been alluded to by many other authors as well, as I continue to discover (e.g., Bachmann, 2000, p. 125; Frith et al., 1999, p. 109).

3 It should also be noted that in addition to correlated non-constitutive activities upstream and downstream from correlated constitutive activities, there can be correlated non-constitutive activities that are *parallel* to correlated constitutive activities. For simplicity, in the arguments that follow I do not consider the parallel category further.

4 Although Chalmers expresses caution over the lesion analysis case, no one would deny that modern understanding of the brain and its functional specialization owes a great debt to such neuropsychological analysis. Phenomenological dysfunction following destruction of certain brain regions by cerebrovascular insult, brain tumors, traumatic brain injury or surgery has informed mechanistic understanding of normal brain function enormously (Damasio and Damasio, 1989; Howieson et al., 2012), as has direct electrical stimulation of the brain (Borchers et al., 2012; see Stepwise Stimulation and Combined Inhibition/Stimulation).

5 There are differences between the terms “basis,” “mechanism”, “substrate,” and “constitution,” however, in most of what follows, I use these terms synonymously. Hence, by “NCnC” I mean to equally imply the neural “basis,” “mechanism,” or “substrate” of consciousness. At times I will state these terms in full to remind the reader of this point and indeed, on this broad construal, one could even “define” the NCnC as the neural basis, mechanism, or substrate of consciousness. However, although the Cr/Cn distinction problem is able to be appreciated and analyzed using any of these broadly synonymous terms, use of “constitution” terminology becomes relevant and informative later, as a specific relation within philosophy of mind (with additional implications for science thereafter). It is because of the relational complexities in the philosophical domain that I resist any further simple definition of the NCnC. For example, a definition could be proposed that states the NCnC is the neural activity that simply *is* the conscious state in question, but any such definition then invites debate about contrasting the “is of identity” with the “is of constitution” (see Miller, 2007, and various papers in Miller, in press-a).

6 On the issue of neuron types, Koch (2004, p. 282) refers to perception-dependent neural activity high in the visual processing pathway (inferior temporal cortex, IT, and superior temporal sulcus, STS) during binocular rivalry as follows:
It is implausible that all of the storied IT and STS neurons that follow the percept express its phenomenological attributes directly. Some must be involved in the underlying winner-take-all operations; others must relay the winner’s identity to the motor centers to initiate behavior or to short-term memory for future recall; some must carry a transient signal indicative of a perceptual switch; and still others might represent the same information but in a delayed manner. When considering the temporal profile of cellular responses in these areas, I am struck by their extreme heterogeneity. An entire menagerie of distinct patterns can be observed. Some cells fire in a transient manner whereas others respond in a more sustained fashion. Some fire in bursts, some show a pronounced, rhythmic discharge in the 4–6 Hz range, while others peak early before settling down to a more sedate and sustained pace of firing. Do these reflect discrete cell types with discrete functions and connectivity patterns? This will be important to know.

7 Bayne and Hohwy (2013; see also Hohwy and Bayne, in press) refer to *differentiating* NCrCs, which are considered neural states that are selectively implicated in particular kinds of conscious states in an already conscious creature.

8 As neurophysiological understanding develops, so too will the notion of what an NCrC block could be taken to be in this analogy—that is, what appropriate “units” of neurophysiological signaling will turn out to be. For now, however, each block represents individual NCrCs such as those being identified at various levels of constituents, organization and processes in studies of binocular rivalry.

9 In my view, and without wishing to undermine or dismiss these important conceptual and methodological efforts, I consider the strategies so far proposed to distinguish NCrC precursors/prerequisites, NCrC proper/substrate, and NCrC consequences by de Graaf and Sack (in press) and Aru et al. (in press) generally fall into this category, appealing to ever more refined means of identifying loosely from tightly correlated neural activities. This refinement certainly helps to close in on the constitution of consciousness but the Jenga analogy begins only *after* application of such strategies.

10 With such a brief treatment here, I do not wish to downplay the importance and complexity of timing in the brain with respect to correlations between neural and phenomenal states. This issue is itself likely to be a source of interpretive difficulty and hence there will be many cases in which such “clear ascription” will not be possible. Indeed, this fact underscores the problems being discussed in the present paper. Nonetheless, in cases for which clear ascription appears possible, timing can be used to screen off candidates for constitutive neural activity. For a detailed source on the issue of timing and phenomenal consciousness, see Bachmann (2000) and for a more recent example of timing studies, see Liu et al. (2009).

11 I provided an example of this fourth category in my first explication of the Cr/Cn distinction problem (Miller, 2001) in terms of the expected correlated neural activity that would be found in subcortical nuclei controlling eye movement tracking (optokinetic nystagmus) during rivalry with drifting gratings. Despite being correlated with consciousness during such rivalry, the known mechanistic function of eye movement nuclei suggests a non-constitutive role for this individual NCrC.

12 Herewith, I combine points (ii), (iii), and (iv) such that when I refer to *tightly* correlated NCrCs, I mean to also include the precise timing and mechanistic function issues.

13 It is important to note that the Jenga analogy is just that—an analogy. It *schematizes* the stepwise inhibition approach to illustrate the points I am making. There is certainly no implied direct equivalence between removing a block from a tower of mechanically related blocks on the one hand, and inhibiting a neuron, neural circuit or set of neural circuits within an exquisitely complex, dynamic and inter-related neural system with the capacity for functional and structural reorganization, on the other. Aside from differences in complexity between a Jenga tower and a neural system, there are further points of difference such as the effect that inhibiting a neuron has on the neuron’s milieu and on non-neural structures like nearby glia and vasculature (Logothetis, 2010). Because of these issues, problems of interpretation can arise with respect to whether it is the neural inhibition that is (or is not) changing consciousness or it is the secondary physiological effects of that inhibition. The same interpretive problems arise regarding neural stimulation (discussed in the next section, where further caveats are raised).

14 This is not the place to discuss in detail the difference between the causation and constitution relation. Suffice to say, I believe the causation relation should be restricted to usage in which one neural activity causes another (hence in the context of causal chain processing) or in the context of mechanistic explanation in which a mechanism or operational part has causal influence on other mechanisms or operational parts (see Craver, 2007 for more on the distinction between causal/etiological and constitutive mechanisms). I do not think the causation relation should be used in the context of the brain–mind relation. That is, brain states constitute mental states, or are identical with them, or realize them, or the relation is one of supervenience, but brain states do not cause mental states. These matters are taken up in much greater detail in various papers in Miller (in press-a). For the view that causation is in fact the relevant brain–mind relation, see Neisser (2012). On the issue of mental causation (i.e., whether mental states could cause brain states and behavior), I do not address this at all, but a constitution, identity, realization or supervenience relation would not preclude mental states causing *other* brain states and consequent behavior—these relations would just preclude mental states causing their *own* brain states.

15 Indeed, Tononi and Koch (2008, p. 257) note in their update of the NCrC program:
The growing ability of neuroscientists to manipulate in a reversible, transient, deliberate, and delicate manner identified populations of neurons using methods from molecular biology combined with optical stimulation enables the intrepid neuroengineer to move from correlation—observing that a particular conscious state is associated with some neural or hemodynamic activity—to causation.
While agreeing with such a sentiment, I would argue that the intrepid neuroengineer should wish to go beyond not just the correlates of consciousness, but also beyond causation (see footnote 14), to ultimately reach the constitution of consciousness.

16 The reverse Jenga strategy could theoretically be applied without a specific factor NCrC map first being identified with recording techniques (though the consequent trial and error task would be far more onerous). Such a scenario might be envisaged if developments in highly precise combined inhibition and stimulation technologies occur more rapidly than developments in highly precise recording technologies. However, this is perhaps unlikely given optogenetics, for example, is not just an inhibition and stimulation technique but is also itself a recording technique. Hence developments in its (safe) recording capacity are likely to occur more or less concurrently with developments in its (safe) inhibitory and stimulatory capacity.

17 It is not clear what it would be like for a subject to be in a state in which all specific factor NCrCs were inhibited but all enabling factor NCrCs remained active, but this is an issue I have side-stepped and is debated elsewhere (Noë and Thompson, 2004; Hohwy, 2009; Hohwy and Bayne, in press; Neisser, 2012). Confusion could also arise if one were to describe non-constitutive causal chain specific factor processes as themselves enabling factors, so this should be avoided. Enabling factors should be restricted to those neural activities present for *all* conscious states in a conscious creature.

18 Neural activity for reporting will thus also need to remain untouched; this of course raises issues of access versus phenomenal consciousness (Block, 1995, 1996; discussed in Klink et al., in press) but again this is not my point here. For more on reporting and downstream NCrCs, see Aru et al. (in press); de Graaf and Sack (in press); Hohwy and Bayne (in press), and van Boxtel and Tsuchiya (in press).

19 In the case of combined inhibition/stimulation (and indeed, only in this case), the minimally sufficient NCrC and the minimally sufficient NCnC can be considered equivalent constructs.

20 Note that in philosophy, multiple realizability refers to functional roles, including consciousness, being realized by *any* substrate, not just neural activity, with such roles thus being substrate-independent. My interest in multiple realizability, however, concerns just *neural* multiple realizability. In Miller (2007), I argued that within the bounds of the problem of direct intersubjective exchange and the Cr/Cn distinction problem, the science of consciousness could nonetheless make progress on issues of neural multiple realizability. As it turns out, such progress was already underway, utilizing the phenomenon of rivalry (Maier et al., 2007). These investigators examined the perception-dependency of single-unit responses to a variety of bistable stimuli and showed, amongst other findings, that the same perceptual state can be correlated with different neural activity patterns in macaque middle temporal (MT) area.

21 Given the redundancy problem (P2), the “closing in” notion may be misleading because isolated stimulation would actually overshoot such closing in, through its exclusion of redundantly constitutive neural activities. Nonetheless, identification of at least some constitutive neural activity is what is meant by “closing in” on the problem.

22 For a detailed analysis of many of the principles I have been discussing from the perspective of philosophy of science, see Craver (2007). Craver’s analysis includes discussion (with specific scientific examples) of notions of mere correlates, interference and stimulation interventions, redundancy, and constitutive relevance (on the latter, see footnote 24). He provides examples of scientific approaches to dealing with redundancy, but not in the context of consciousness.

23 The arguments I have presented thus far have considered the terms neural “basis,” “mechanism,” “substrate,” and “constitution” of consciousness as synonymous, but in the context of mechanistic explanation, the term “mechanism” has its own implications (just as the “constitution” term has its own implications also; see later).

24 The notion of a mereology of processes differs from the mechanistic approach to activities in a subtle but potentially important way. Craver (2007, p. 136) notes, “There are no mechanisms without active organisation, and no mechanistic explanation is complete or correct if it does not capture correctly the mechanism’s active organisation.” Craver’s notion of activities (Machamer et al., 2000; Craver, 2007) is one with a decidedly mechanistic flavor. In addition, Craver (2007) outlines the importance of attending to the notion of *constitutive relevance* in mechanistic explanation (i.e., identifying relevant components and their interactions and excluding irrelevant components and their interactions). While this is no doubt indeed important for mechanistic explanation—and for finding constitutive mechanisms of consciousness (as sought by Revonsuo, 2006)—understanding the constitution of consciousness may need to take more than just a mechanistic explanation form. Indeed, this is why the title of this paper—“Closing in on the Constitution of Consciousness”—stands in contrast to the title of a chapter in Revonsuo (2006)—“Closing in on the Mechanisms of Consciousness.” Although the mechanistic explanation form may turn out to be the best way by which to understand the constitution of consciousness, mereological approaches, in my view, also need developing. This will not, however, be easy territory. Craver’s (2007) use of the notion of “constitutive explanation” (used in the mechanistic context) is not intended to have metaphysical relational implications. Thus he notes (p. 20), “Metaphysicians reserve the term “constitutive” for a specific relation that has more entanglements than I intend.” A metaphysical mereological notion of “constitutive” may well, I suggest, have different implications for the notion of constitutive relevance than the mechanistic notion of “constitutive.” Finally, in answering how we might test hypotheses regarding which microconstituents can be considered part of the NCnC and which not (hence which are constitutively relevant), microconstituent stepwise inhibition could be applied, with assessment of whether a conscious state changes or does not change accordingly. However, drawing conclusions from such interventions, and determining whether a Cr/Cn distinction problem also exists at this level (see Miller, in press-b), would seem to require the combination of mereological and mechanistic analyses that I am advocating.

25 An abbreviated, conjoined and simpler set of claims, without specific reference to empirical strategies and to P1–P3, would be: C1: The ultimate aim of consciousness science is not to identify the minimally sufficient neural correlates of consciousness, but rather to identify the (whole) neural basis, mechanism, substrate, or constitution of consciousness. C2: The current foundational construct of consciousness—the minimally sufficient neural correlates of consciousness—can pick out a different neural activity set to that picked out by the neural basis, mechanism, substrate, or constitution of consciousness. Consciousness science can nonetheless continue to work toward identifying the minimally sufficient neural correlates of consciousness. C3: Although there may be empirical strategies to identify some constitutive neural activity, there is not yet evident a strategy to identify the whole neural constitution of consciousness. Empirical approaches to this problem need to be developed, and may require entirely new scientific strategies.
